# Sixty years of community change in the prairie–savanna–forest mosaic of Wisconsin

**DOI:** 10.1002/ece3.4251

**Published:** 2018-07-28

**Authors:** Laura M. Ladwig, Ellen I. Damschen, David A. Rogers

**Affiliations:** ^1^ Department of Integrative Biology University of Wisconsin – Madison Madison Wisconsin; ^2^ Biology Department University of Wisconsin – Parkside Kenosha Wisconsin

**Keywords:** disturbance, fire, grassland, historic vegetation surveys, species loss, succession, understory, woodland

## Abstract

Biodiversity loss is a global concern, and maintaining habitat complexity in naturally patchy landscapes can help retain regional diversity. A mosaic of prairie, savanna, and forest historically occurred across central North America but currently is highly fragmented due to human land conversion. It is unclear how each habitat type now contributes to regional diversity. Using legacy data, we resurveyed savanna plant communities originally surveyed in the 1950s to compare change in savannas to that in remnant forests and prairies. Savanna community structure and composition changed substantially over the past 60 years. Tree canopy density nearly doubled and many prairie and savanna specialist species were replaced by forest and non‐native species. All three habitats gained and lost many species since the 1950s, resulting in large changes in community composition from local colonizations and extinctions. Across all three habitats, regional species extinctions matched that of regional colonization resulting in no net change in regional species richness. Synthesis—Despite considerable species turnover within savannas, many species remain within the broader prairie–savanna–forest mosaic. Both regional extinctions and colonizations were high over the past 60 years, and maintaining the presence of all three community types—prairie, savanna and forest—on the landscape is critical to maintaining regional biodiversity.

## 1 INTRODUCTION

Biodiversity loss is a global concern (Chapin et al., 2000; Rockström et al., 2009; Steffen et al., 2015) largely caused by humans. Increased habitat fragmentation decreases total habitat area and connectivity between remnant habitat patches, thus accelerating regional extinctions (Alstad & Damschen, 2015; Haddad et al., 2015; Krauss et al., 2010; Rogers, Rooney, Hawbaker, Radeloff, & Waller, 2009). Human alterations to historic disturbances regimes, such as decades of no fire, can trigger ecosystem shifts and further species loss (Alstad et al., 2016; DeSantis, Hallgren, & Stahle, 2011; Li & Waller, 2015; Nowacki & Abrams, 2008; Umbanhower, 1996). Changing climate, including a greater frequency of extreme climate event and warmer temperatures (IPCC 2014), can alter the pace and direction of community change (Walther, Beißner, & Burga, 2005). Furthermore, these forces act within landscape contexts that also influence ecological processes (Tscharntke et al., 2012). Environmental heterogeneity can help create and maintain high biodiversity (Pearson, Turner, Gardner, & O’Neill, 1996); therefore, retaining heterogeneous habitats can be beneficial for keeping species on the landscape.

One naturally heterogeneous system in both space (Davis, 1977; Hanson, 1922) and time (Baker et al., 2002) is the prairie–savanna–forest mosaic within the central U.S.A. (Anderson, 1983). The shifting, patchy transition between open prairie and closed canopy forest contains sites along a continuum of tree canopy densities and understory compositions. Within the mosaic, even small patches of habitat can contain substantial diversity (Simberloff & Gotelli, 1984), and the ecotones among habitat types host unique species (Williamson, 1975). In particular, savannas represent a mid‐point between open grassland and closed forest and therefore support species from both habitat types as well as savanna specialists to enhance local and regional biodiversity (Bray, 1960; Curtis, 1959). For example, the prairie–savanna–forest mosaic historically occurred throughout southern Wisconsin (Curtis, 1959; Leitner, Dunn, Guntenspergen, Stearns, & Sharpe, 1991; Transeau, 1935), and these savannas have diverse, forb‐dominated understories (Leach & Givnish, 1999). Species composition is determined by light availability (Bray, 1958; Leach & Givnish, 1999; Pavlovic, Grundel, & Sluis, 2006), disturbance history (Weiher, 2003) and soil properties (Leach & Givnish, 1999; Weiher, 2003). Given that savannas provide a variety of microsites amenable to species from prairies and forests, savannas may promote regional species persistence under global change and be critical to retaining biodiversity on the landscape.

The natural habitat complexity that supports biodiversity throughout the prairie–savanna–forest mosaic has become increasingly compromised as humans alter the landscape (Pogue & Schnell, 2001). Land conversion to agriculture and urban expansion following European settlement greatly decreased coverage of natural systems (Anderson, Fralish, & Baskin, 1999; Pogue & Schnell, 2001; Rhemtulla, Mladenoff, & Clayton, 2007). By the 1950s, prolonged, widespread livestock grazing helped make oak savannas with intact understory communities the rarest natural ecosystems in Wisconsin (Curtis, 1959). Furthermore, altered disturbance regimes, namely decreased fire frequency leading to mesification (Nowacki & Abrams, 2008) and increased deer herbivory (Wiegmann & Waller, 2006), caused substantial compositional shifts in recent decades (Rogers, Rooney, Olson, & Waller, 2008). Within forest understories, plant communities homogenized taxonomically over the past several decades (Rogers et al., 2008). Within prairies, loss of fire and increased isolation of remaining patches accelerated local extinctions and the spread of woody and weedy species at the cost of prairie specialist species (Alstad et al., 2016; Kraszewski & Waller, 2008; Leach & Givnish, 1996). Historically, savannas contained a mix of both forest and prairie species (Bray, 1960) and could play a critical role in maintaining biodiversity throughout the region. Given the substantial change to regional forests and prairies (Alstad & Damschen, 2015; Alstad et al., 2016; Rogers et al., 2008), savannas presumably also changed, but the change has not been evaluated.

Here, we use a unique legacy dataset from remnant savanna sites to ask: (Q1) How has the species composition of savanna communities changed over the past 60 years, and how does the amount of change compare to that of prairies and forests? (Q2) Have savannas acted as a refuge for prairie and forest species over the past 60 years to help maintain regional biodiversity? (Q3) How does the relative contributions of local diversity in prairies, savannas, and forests contribute to regional biodiversity?

## 2 MATERIALS AND METHODS

### 2.1 Study area and historic data

**Figure 1:**
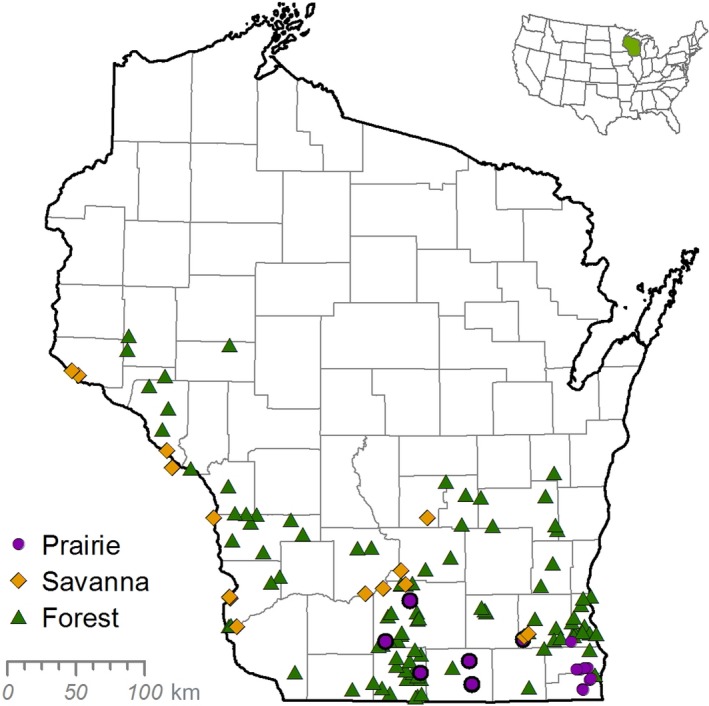
Survey locations of forest (triangles), savanna (diamonds), and prairie (circles) sites across Wisconsin

From 1951 to 1954 (hereafter referred to as the 1950s), Roger Bray and John Curtis surveyed remnant savannas across southern Wisconsin (42 – 45° N, 88 – 93° W; Bray, 1960; Figure 1) as part of larger project to classify the vegetation of Wisconsin (Curtis, 1959; Waller, Amatangelo, Johnson, & Rogers, 2012). Sites included both oak savannas and cedar glades that represented the best remaining native savannas in the state. Care was taken to select sites with minimal human disturbance, including limited logging and grazing and intact native understory communities not heavily invaded by non‐native species (Bray, 1955, 1960).

### 2.2 Site relocation and vegetation surveys

In 2014, original survey locations of savannas were relocated and surveyed following the same methods from the 1950s surveys. Resurvey effort focused on sites with species frequency data (as opposed to only species presence) for understory communities in 1950s and sites that were not converted to a different land‐use type since the 1950s (e.g., pasture, tree plantation, golf course).

To relocate the original survey sites, detailed notes and hand‐drawn maps from the 1950s surveys were compared to aerial images and maps in ArcGIS (ESRI, Redlands, CA, USA). Specifically, we used historical aerial images obtained from the UW Geography Museum, current aerial images, Wisconsin Land Economic Inventory (Bordner Survey) maps from the 1920s (http://digital.library.wisc.edu/1711.dl/EcoNatRes.WILandInv), and topography from USGS (http://store.usgs.gov). Additionally, tree density in historic images was compared to tree density in survey records to further locate transect placement within a site. In most cases, current land use was detectable from contemporary aerial images, but for sites where land use was uncertain, additional land‐use maps were obtained from the Southeastern Wisconsin Regional Planning Commission (http://www.sewrpc.org). A polygon was drawn around the extent of each intact site, and parallel transects were drawn within the site to guide plot location during field surveys. The ideal plot layout was a grid with sample points 10 m from one another, but site area and dimensions restricted transect configuration at most sites. To obtain land‐use histories for the past 60 years, we talked with landowners and specifically asked whether and when the site had been grazed, burned, harvested, or experienced any other disturbance since the original survey. Signs of recent disturbances, including stumps and burn scars, were noted during resurveys.

In summer 2014, we surveyed the savanna communities following the same methods as the initial 1950s survey (Bray, 1960). We selected at least 20 sampling points >10 m from each other and surveyed the canopy, understory, and shrub vegetation at each point. For canopy trees, the random pairs technique was used (Cottam & Curtis, 1949), and two trees were measured at each point, including the size (DBH), species identity, and distance between trees. For understory vegetation, a 1 × 1 m quadrat was placed North and center of the sampling point and presence of all woody species less than 1 m tall and all herbaceous species rooted in the quadrat were recorded. First‐year tree seedlings were recorded separately from older seedlings given the high mortality rate of first‐year seedlings. For the shrub layer, a straight 2‐m‐wide transect was positioned between the two surveyed trees. All shrubs >1 m tall and small trees (>1 m tall and <10 cm DBH) rooted within the transect were identified to species and counted.

Taxonomic resolution was kept consistent between the 1950s and contemporary surveys. Most plants were identified to species, but some were identified to genus (e.g., *Carex* spp.) as per the original sampling. Nomenclature followed the Wisconsin Flora (Chadde, 2013).

In total, sixteen savanna sites were resurveyed in 2014, four of which were previously resurveyed in 2004 (Mills, 2008). All sites experienced altered disturbance regimes, especially through the loss of fire. In recent decades, three sites experienced substantial management including canopy thinning and the return of prescribed fire. To compare changes in savannas relative to those in prairies and forests, savanna resurvey data were compared to forest and prairie resurvey data from similar historic datasets (Curtis, 1955; Rogers et al., 2008; Alstad & Damschen, 2015; Figure 1). Hardwood forests were resurveyed from 2002 to 2004 (Rogers et al., 2008), and prairies were resurveyed in 2012 (Alstad & Damschen, 2015). We also included resurvey data from eleven additional prairie sites that were initially sampled using identical methods to the surveys above (Whitford, 1958) and resurveyed in summer 2015.

### 2.3 Data analysis

To examine changes in savanna plant communities over the past 60 years, we evaluated compositional change for both the canopy and understory. First, we compared changes in canopy tree density (tree per acre) between 1950s and 2014 with a paired *t* test. Canopy composition at the two time points was compared with a nonmetric multidimensional scaling (NMDS) approach using Bray–Curtis dissimilarity. To test for a significant difference between canopy composition in the 1950s and 2014, we used the PERMANOVA function “adonis” in the “vegan” package in R. Except where noted elsewise, all statistical analysis was run in R (R Foundation for Statistical Computing, Vienna, Austria, version 3.2.1). Second, changes in savanna understory communities were assessed by comparing rank abundance curves from the 1950s and 2014. To visualize changes through time, species were color‐coded based on their presence in savannas and the broader mosaic at both survey points. To determine the degree of change in savannas relative to prairies and forests, we used two separate NMDS analysis, one examining species presence–absence with a Jaccard distance metric and a second examining species abundance with Bray–Curtis dissimilarity. To test for significant differences among habitats types (prairie, savanna, forest), time periods (1950s, 2010s), and their interaction, we ran repeated‐measures PERMANOVAs in PRIMER (Clark & Gorley, 2015). To test whether sites within each habitat type became more similar to one another over time, we ran a multivariate homogeneity of group dispersions (variances) analysis with the function “betadisper” in the “vegan” package to compare the pairwise distance among all sites for each time period. The test is sensitive to sample size, which varied between habitat types, so a separate test was run for each habitat type to individually evaluate changes in homogeneity through time.

To further examine changes in regional diversity throughout the mosaic, we first compared changes in species abundance in savannas relative to neighboring communities and then examined the relative influence of local diversity of each habitat on regional diversity. To determine whether savannas acted as refuges for prairie or forest species, we assessed the ability of savanna sites to retain or gain species that were declining in prairies and forests. To do this, we calculated an index of change for each species found in savannas, specifically:

$$\Delta \;\text{index}=\frac{{\left(f_{2010}-f_{1950}\right)}^{2}}{\left(f_{2010}-f_{1950}\right)}$$

where “*f*” is the count of sites a species occupied during the survey time noted in subscript. If the species decreased between 1950 and 2010, the index was multiplied by ‐1 to differentiate increasing and decreasing species. To test whether savannas acted as a refuge for forest or prairie species, indices of species occurring in forests or prairies were compared with two separate regressions, one for each habitat type. A regression line with an intercept >0 indicated that overall savannas acted as a refuge, while an intercept <0 indicated that either prairies or forests acted as a refuge for savanna species. Next, to understand how relative contributions of local diversity in prairies, savannas, and forests contributed to regional diversity we examined the number of shared and unique species among the three habitat types in both the 1950s and 2010s. Specifically, we examined how many species entered (colonization) and left (extinction) over the past 60 years and whether colonizations and extinctions were limited to one habitat but not the whole mosaic (local) or pertained collectively to all three habitat types (regional).

## 3 RESULTS

**Figure 2:**
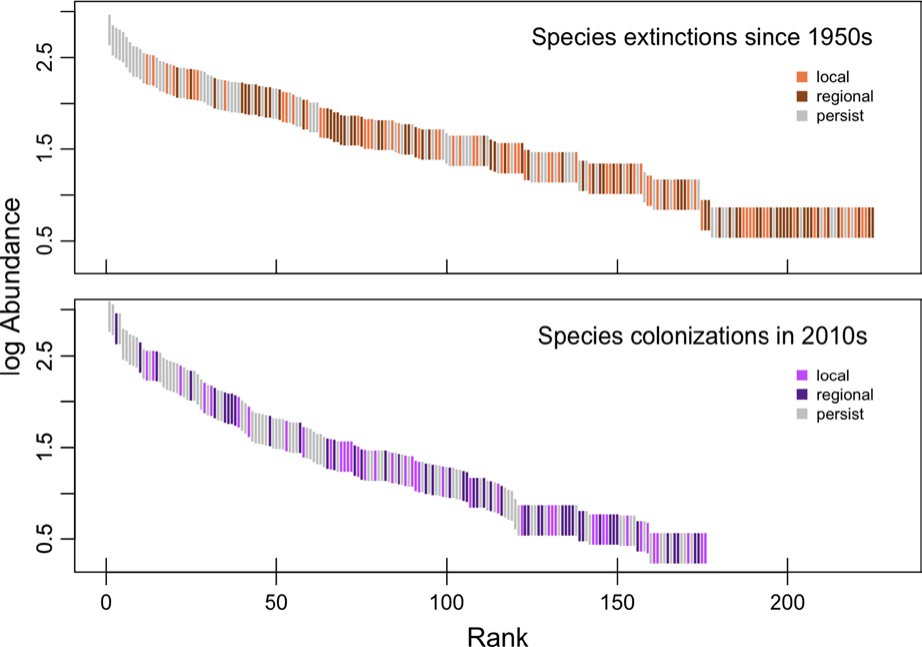
Rank abundance curves of understory species collectively across the 16 savanna sites in 1950s (top) and 2010s (bottom). Each line represents a species and is color‐coded based on its presence during both survey times. Gray indicates species present at both time points (persist), orange/brown indicates species only present in the 1950s (extinction), and purple indicates species only present in the 2010s (colonization). Changes in occurrence (extinctions and colonizations) could be restricted to only savannas and not the whole mosaic (local; lighter color) or could be relate to all habitats in the mosaic (regional; darker color)

Savanna canopy structure changed considerably between the 1950s and 2014 as the number of trees per acre nearly doubled, increasing from 111 ± 17 to 206 ± 22 (*p* = 0.002). Community composition also changed, and the most abundant species in the 1950s (*Juniperus virginiana*) decreased in relative abundance as the abundance of several more mesic species (e.g., *Acer* spp.) increased (Supporting information Figure S1). Although relative abundance of canopy species shifted, overall composition did not change between the 1950s and 2014 based on PERMANOVA results (*p* = 0.1; Supporting information S1). Savanna understory communities also changed considerably. Many species disappeared from savannas and collectively across all sites, species richness decreased from 224 to 175 taxa (Figure 2). In total, 61% of the species present in 1950 (136 of 224) were no longer present in savannas by 2014. Many species that left the systems were prairie or savanna specialist species. Additionally, species that were common in the 1950s become rare or completely absent from contemporary savannas. For example, the five most abundant taxa in the 1950s (*Euphorbia corollata, Poa* spp*., Andropogon gerardii, Amorpha canescens, Schizachyrium scoparium*) once collectively comprised 17% of understory cover across savannas, but presently only cover 0.4% of contemporary savannas. Meanwhile, many new taxa entered savannas over the past 60 years. In total, 50% of the taxa in contemporary savannas (87 of 175) were not present in the 1950s surveys. The colonizing species were mainly forest or non‐native species. Additionally, several less‐abundant species in the 1950s increased through time. The five taxa that increased the most over the past 60 years (*Parthenocissus quinquefoia, Rhamnus cathartica, Carex* spp*., Circaea lutetiana, Zanthoxylem americanum*) once collectively occupied 3% of the total understory vegetation cover across all savannas in the 1950s, but now account for 28% of cover in contemporary savannas.

**Figure 3:**
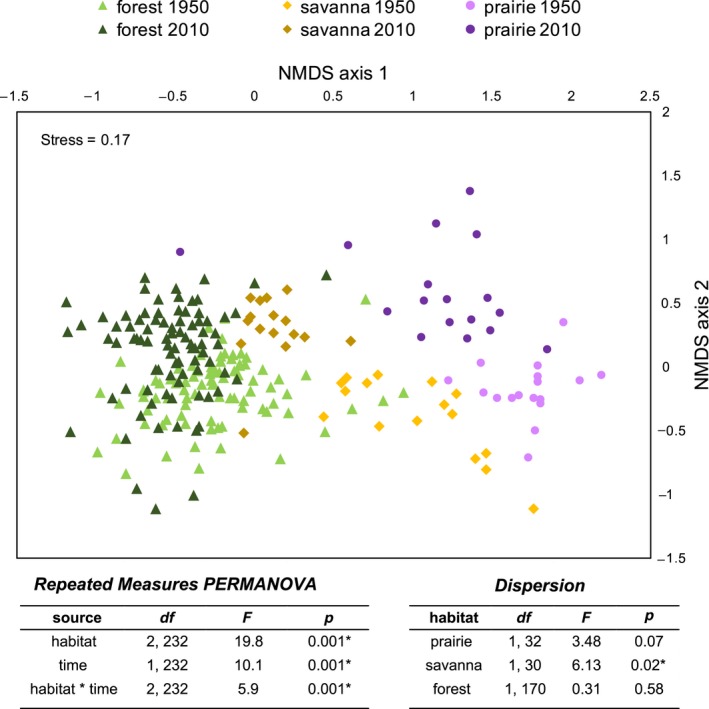
A NMDS of species presence in the 1950s (light) and 2010s (dark) within forest understories (triangles), savanna understories (diamonds), and prairies (circles). Each point represents a plant community at a single site and time. Statistical results from a repeated‐measures PERMANOVA testing whether plant communities differed with regard to habitat type, time, and their interaction, and analysis of multivariate homogeneity of group dispersion testing whether communities testing for changes in variance within each habitat type

All habitat types within the prairie‐savanna‐forest mosaic significantly changed over the past 60 years. Based on the repeated‐measures PERMANOVA comparing species presence/absence in habitats at both time points, communities were statistically different with regard to time, habitat type (savanna, prairie, forest), and the interaction between the two (Figure 3), and pairwise comparisons indicated that all three habitat types were different from one another (*p* = 0.001). Community dispersion among prairie and forest sites did not statistically change over the past 60 years (Figure 3), but savanna sites homogenized and became more similar to each other over the past 60 years (Figure 3). Examining community changes based on species abundance with Bray–Curtis dissimilarity resulted in similar results (Supporting information Figure S2).

**Figure 4:**
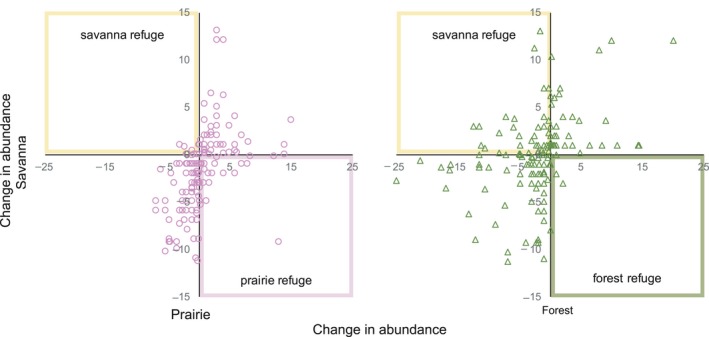
Change in frequency (∆ index; see methods for calculation) of species in prairies (left) and forests (right) relative to savannas between the 1950s and 2010s. Positive changes indicate increased abundance through time while negative changes indicated decreasing abundance. Points within the colored square outlines indicate species that decreased in one habitat but increased in a neighboring, or refuge, habitat

Community change in savannas was often related to change in prairies and forests. Many species that were new to savannas in the 2010s were present in the forests of the 1950s (44%, 39 of 88). Likewise, many taxa that disappeared from savannas were present in the 2010s prairies (41%, 56 of 137; Figure 2b). Change in species abundance of shared species was often similar for both savannas and prairies (*p* < 0.0001, *r* ^2^ = 0.21, *F*_1,167_ = 46.63) and savannas and forests (*p* < 0.0001, *r* ^2^ = 0.12, *F*_1,178_ = 25.67; Figure 4). For example, of the taxa found in both savannas and prairies, 41% (70 of 169) decreased in both habitats. Likewise, 42% (76 of 180) of the taxa shared between savannas and forests decreased through time in both habitats (Figure 4). Although, the direction of change in savannas and forests or prairies was not consistent for all species, some habitats provided a refuge. Specifically, 15% (26 of 169) of taxa decreased in savannas and increased in prairies, and 24% (44 of 180) of taxa decreased in forests and increased in savannas (Figure 4). To a limited degree, prairies acted as a refuge for savanna species, as the intercept of the regression between change in those habitats was significantly lower than 0 (intercept estimate −1.68, *p* < 0.001), but there were no refuge habitat type between forests and savannas (intercept *p* = 0.2).

**Figure 5:**
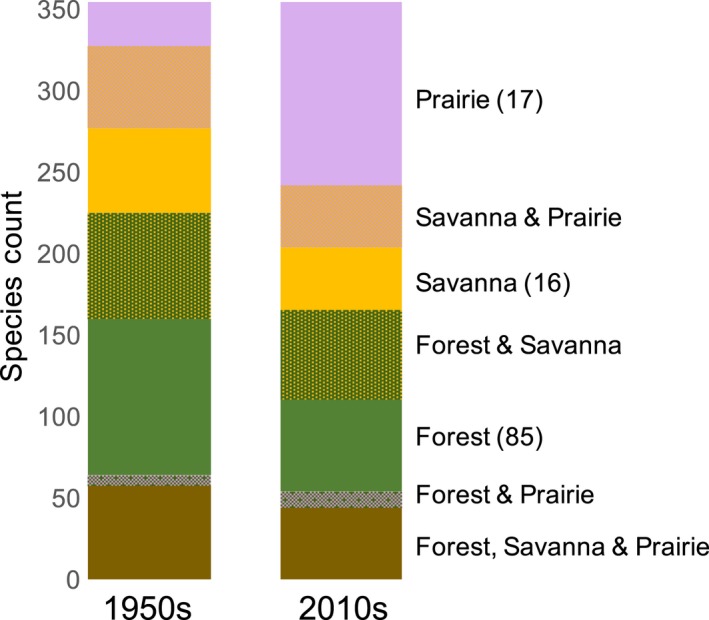
Species richness partitioned among habitat types in the prairie–savanna–forest mosaic in the 1950s and 2010s. Sampling intensity was consistent between times but varied among habitats, as more forest sites (85) were surveyed than prairie (17) or savanna (16) sites

**Figure 6:**
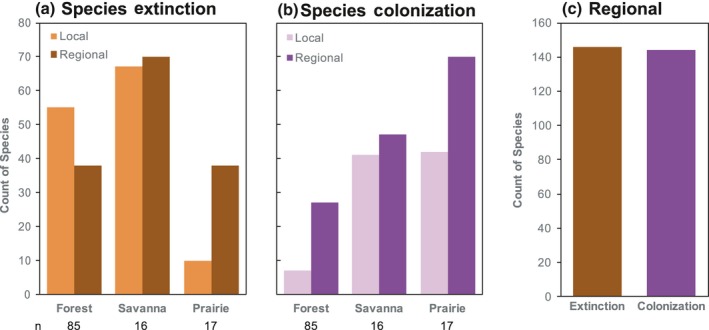
Local (a, b) and regional (c) species colonizations and extinctions in the prairie–savanna–forest mosaic of southern Wisconsin over the past 60 years. The number of sites surveyed (*n*) varies among habitat types

Between the 1950s and 2010s, regional species richness—the collective number of species across all three habitats—remained constant (Figure 5) yet local richness changed in all habitat types (Figure 6). Savannas experienced the greatest species losses over the past 60 years (Figure 6a), and nearly half the species lost from savannas (67 of 137) can currently be found in forests or prairies while half have gone regionally extinct. Species colonizations were highest overall in prairies (Figure 6b), and 62% (70 of 112) of species colonizing prairies over the past 60 years were completely new to the region. Across all habitats, the number of regional extinctions and colonizations was very similar (Figure 6c), leading to no overall change in regional species richness between the 1950s and 2010s (Figure 5). Importantly, our measures of species richness are more comprehensive in forests, as sampling intensity was much greater in forests (85 sites) than in savannas or prairies (16 and 17 sites, respectively).

## 4 DISCUSSION

A mix of prairie, forest, and savanna specialist species once co‐occurred in the savannas of southern Wisconsin but over the past 60 years, sites lost many prairie and savanna species and now more closely resemble forests. Over half the species historically found in both savannas and prairies decreased in abundance over the past 60 years (Figure 4). During this time, fire was largely absent from both savannas and prairies (Alstad & Damschen, 2015; *Ladwig* personal comm.). Species diversity in savannas relates to fire frequency (Peterson & Reich, 2008; Weiher, 2003), and as fire frequency decreased so did the diversity of prairie species. Loss of fire also influenced canopy dynamics, as the most visually apparent change in savannas was a near doubling of tree density since the 1950s (Supporting information Figure S1). Prior to the original surveys, tree density had already increased in savannas since pre‐European settlement (Cottam, 1949) and this trend continued over the past 60 years. One factor contributing to the continued increase in tree density, particularly of mesic tree species, may be the loss of historically routine, low‐intensity fires that maintained savanna ecosystems (Wolf, 2004). In the absence of fire, trees and shrubs can more easily establish and succession progresses (Nowacki & Abrams, 2008; Wolf, 2006). Although savannas changed substantially, the observed changes were expected given the loss of fire and increase in woody plant cover. An increase in woody plant cover is not only a measure of change but also a driver of change (Briggs et al., 2005). Across North American grasslands and savannas, plant diversity decreases as woody plant cover increases (Rataczjak, Nippert, & Collins, 2012). As the savanna canopy closed, understory light availability decreased and presumably contributed to the large loss in prairie species. Regionally, the tall, closed canopy structure of forests and open, herbaceous‐dominated structure of prairies remain, but the structure of savannas—patchy canopy with dense herbaceous understory—is largely lost from the natural landscape. The intermediate canopy structure of savannas provides suitable microsites for both forest and prairie species, allowing for heightened biodiversity in a small area (Leach & Givnish, 1999), but this benefit for biodiversity is now missing in the region.

All three habitats within the prairie‐savanna‐forest mosaic experienced large ecological shifts over the past 60 years (Rogers et al., 2008; Alstad et al., 2016; Figure 3). Woody and non‐native species increased in both forest understories and prairies (Alstad & Damschen, 2015; Rogers et al., 2008) and similar changes occurred in savannas (Supporting information Figures S1 & S2). Meanwhile, many prairie and savanna specialist species decreased or went locally extinct over the past 60 years (Alstad et al., 2016; Figures 2 and 6). At a regional scale, the number of species colonizations and extinctions was roughly equal (Figure 6c), leading to no overall change in regional species richness (Figure 5). In a previous study of 47 prairies in the region, community composition also changed substantially over the past 60 years, but extinctions were greater than colonizations (Alstad et al., 2016). Differences in sampling intensity likely influence the patterns observed, particularly if most species extinctions are of less‐common species that require a higher sampling intensity to capture and most species colonizations are weedy, generalist species present at many sites. Here, sampling intensity was greater in forests (85 sites) than in either savannas (16 sites) or prairies (17 sites) and likely influenced species richness measures. But regardless of sampling intensity, large shifts in community composition occurred in all three habitats in the prairie‐savanna‐forest mosaic (Figure 3, Rogers et al., 2008; Alstad et al., 2016) and the plant communities present today are much different from those 60 years ago.

The prairie‐savanna‐forest mosaic has been dynamic in the past (Davis, 1977) and will likely continue to shift in the future. The absence of fire may intensify mesification (Nowacki & Abrams, 2008) and promote woody encroachment in open areas (Heisler, Briggs, & Knapp, 2003; Van Auken, 2000), favoring forests over prairies. Alternatively, larger droughts that stress trees may favor prairies and savannification of forested areas (Allen, Breshears, & McDowell, 2015; Brzostek et al., 2014; Frelich & Reich, 2010; Gustafson & Sturtevant, 2013). Maintaining the full variety of habitats within the prairie‐savanna‐forest mosaic could allow for future retention of species on the landscape as species continue responding to global change.

The gradient of community types once present throughout the prairie‐savanna‐forest mosaic is disappearing, but not yet gone. In the 1950s, savannas were already rare on the landscape (Curtis, 1959) and contained a mix of prairie to forest species (Bray, 1960; Figure 3). Sixty years later, the occurrence of savanna specialist and prairie species in savannas is rare, as understories now more closely resemble forests (Figure 3). Although the abundance of prairie and savanna specialist species has greatly decreased in recent decades, many species still remain in savannas but at much lower densities (e.g., occurring in one quadrat at one site), and some of these species could act as indicator species to predict restoration success (González, Rochefort, Boudreau, & Poulin, 2014). Yet it remains unknown how long savanna understory species can persist without frequent fire, making it urgent to restore remnant savannas. Returning historic disturbance regimes (e.g., periodic low fire; Peterson & Reich, 2001, 2008; Weiher, 2003; Weiher & Howe, 2003) and initiating management techniques to reduce canopy cover (grazing, Hedtcke, Posner, Rosemeyer, & Albrecht, 2009; thinning, Brudvig & Asbjornsen, 2009) could allow savanna species composition (Bowles, Apfelbaum, Haney, Lehnhardt, & Post, 2011) and function (Brudvig & Asbjornsen, 2009) to return. Our data suggest there may still be time to revitalize remnant savanna sites and the prairie‐savanna‐forest mosaic, but the time is now.

## CONFLICT OF INTEREST

None declared.

## AUTHOR CONTRIBUTIONS

LML, EID, and DAR conceived the ideas, designed the methodology, and collected data; LML analyzed the data; LML, EID, and DAR contributed critically to the drafts.

## DATA ACCESSIBILITY

Data will be available through the Dryad Digital Repository (datadryad.org) and historical data are also available through the UW Plant Ecology Laboratory (botany.wisc.edu/PEL/data.html).
