# Supplementary material for: Sixty years of community change in the prairie–savanna–forest mosaic of Wisconsin
Source: Ecol Evol. 2018 Jul 28;8(16):8458–66. doi: 10.1002/ece3.4251 (PMC6145032; doi:10.1002/ece3.4251)
Supplement: Supplementary file 1 [file ECE3-8-8458-s001.docx]

**Supplemental Material**

Figure S1. A NMDS of savanna canopy communities in 1950s and 2014 (upper). Each arrow is a separate site and connects the 1950s and 2014 centroid location for that site. Mean relative basal area of all canopy species present in savannas (lower).

Figure S2: A NMDS with Bray-Curtis dissimilarity of species abundance in the 1950s (light) and 2010s (dark) within forest understories (triangles), savanna understories (diamonds) and prairies (circles). Each point represents a plant community at a single site and time. Statistical results from a repeated measures PERMANOVA testing if plant communities differed with regard to habitat type, time and their interaction, and analysis of multivariate homogeneity of group dispersion testing if communities testing for changes in variance within each habitat type.
